# Neoadjuvant immune checkpoint inhibition in the management of glioblastoma: Exploring a new frontier

**DOI:** 10.3389/fimmu.2023.1057567

**Published:** 2023-02-17

**Authors:** Stephen C. Frederico, Corbin Darling, John P. Bielanin, Alexandra C. Dubinsky, Xiaoran Zhang, Constantinos G. Hadjipanayis, Gary Kohanbash

**Affiliations:** ^1^ University of Pittsburgh School of Medicine, Pittsburgh, PA, United States; ^2^ Department of Neurological Surgery, University of Pittsburgh, Pittsburgh, PA, United States

**Keywords:** glioblastoma, GBM, immunotherapy, ICI, neoadjuvant, brain

## Abstract

Brain tumors are one of the leading causes of cancer related death in both the adult and pediatric patient population. Gliomas represent a cohort of brain tumors derived from glial cell lineages which include astrocytomas, oligodendrogliomas and glioblastomas (GBMs). These tumors are known to grow aggressively and have a high lethality with GBM being the most aggressive tumor in this group. Currently, few treatment options exist for GBM outside of surgical resection, radiation therapy and chemotherapy. While these measures have been shown to marginally improve patient survival, patients, especially those diagnosed with GBM, often experience a recurrence of their disease. Following disease recurrence, treatment options become more limited as additional surgical resections can pose life threatening risk to the patient, patients may be ineligible for additional radiation, and the recurrent tumor may be resistant to chemotherapy. Immune checkpoint inhibitors (ICIs) have revolutionized the field of cancer immunotherapy as many patients with cancers residing outside the central nervous system (CNS) have experienced a survival benefit from this treatment modality. It has often been observed that this survival benefit is increased following neoadjuvant administration of immune checkpoint inhibitors as tumor antigen is still present in the patient which enables a more robust anti-tumor immune response. Interestingly, results for ICI-based studies for patients with GBM have been largely disappointing which is a stark contrast from the success this treatment modality has had in non-central nervous system cancers. In this review, we will discuss the various benefits of neoadjuvant immune checkpoint inhibition such as how this approach reduces tumor burden and allows for a greater induction of an anti-tumor immune response. Additionally, we will discuss several non-CNS cancers where neoadjuvant immune checkpoint inhibition has been successful and discuss why we believe this approach may provide a survival benefit for GBM patients. We hope this manuscript will foster future studies aimed at exploring whether this approach may be beneficial for patients diagnosed with GBM.

## Introduction

Brain tumors are now the leading cause of cancer related death in males aged 39 years and below, and females aged 19 years and below (1). Gliomas are one of the drivers of brain tumor mortality as these tumors are known to grow aggressively and respond poorly to chemoradiation therapy (2). Gliomas arise from various glial cell types, whose normal function is to support neurons within the brain and include tumors defined as astrocytomas, oligodendrogliomas, and glioblastomas (GBMs) (2, 3). Oligodendrogliomas are characterized by 1p/19q chromosomal codeletion which is accompanied by an IDH mutation (4). The median survival time for those diagnosed with an oligodendroglioma is 10-12 years, with 5-year progression-free (PFS) and overall survival (OS) rates of 51-83% (5–8). IDH mutant astrocytomas, often do not boast as good of a prognosis as these tumors are known to grow more aggressively as compared to oligodendrogliomas (4). GBM which is classified as an IDH wildtype tumor is the most common glioma and associated with a dismal prognosis (4). The five-year survival for patients diagnosed with GBM aged 20-44, 45-54 and 55-64 is 22%, 9%, and 6% respectively (9). The current standard of care for patients diagnosed with GBM includes surgical resection, chemotherapy, and radiation therapy (10). However, these treatments often induce a host of negative side effects for patients and only marginally improve a patient’s OS.

Multiple factors contribute to GBM having such a high lethality. The first is that GBMs typically grow in a diffuse manner and infiltrate the surrounding brain (11). This makes it nearly impossible for surgeons to completely resect the tumor as tumor cells reside beyond the main tumor bulk mass. These infiltrative tumor cells are difficult to visualize during surgery and are also difficult to be imaged by MRI. The inability to achieve a full resection of the tumor often leads to tumor recurrence within months after initial surgery and the ultimate demise of GBM patients (11).

A second contributor to GBM progression is the immunosuppressive nature of the tumor microenvironment (TME) (12) (13). Specifically, GBM overexpresses immune inhibitory proteins such as ICAM-1 which interacts with LFA-1 and enables myeloid derived suppressor cell (MDSC) accumulation within the TME (14). MDSCs suppress anti-tumor T-cells through the expression of anti-inflammatory molecules such as TGF-beta and arginase (15). GBMs also express Fas ligand, CD70, as well as PD-L1 (16). These molecules result in either T-cell death or T-cell inhibition. It has also been well documented that there are a limited number of T-cells that are present within the TME. T-cells that are present within the TME are typically classified as exhausted and may even function as immune suppressors (17). This immunosuppression is only amplified in GBM patients as most patients receive dexamethasone to control cerebral edema (18). Dexamethasone has been shown to upregulate immunosuppressive checkpoint molecules such as CTLA4 which inhibits T-cell anti-tumor activity (18). Additionally, dexamethasone has been shown to lead to an overall reduction in T-cell proliferation (18).

Intertumoral heterogeneity is an additional contributor to GBM progression as some studies have shown that 50% of recurrent GBM samples share only half the genetic mutations that were housed in the original tumor (19). Studies by Soeda and colleagues were some of the first to show that GBM subclones, derived from a single patient, had distinct cell populations and the sensitivity of each subclone to an inhibitor of epidermal growth factor receptor were dissimilar (20, 21). Later studies by Dirkse and colleagues expanded upon this work, by revealing that phenotypic heterogeneity that is observed in GBM, is derived from cancer stem cells undergoing reversible state transitions that are instructed by the tumor microenvironment (22). Additionally, it has been observed that the anatomical location of GBM within different sites of the brain, impacts the mutational landscape of the tumor (23).

A final contributor to GBM progression is the blood brain barrier (BBB) (24). The BBB is a complex of tight junctions joining endothelial cells which prevent most substances from passing into the cranial vault (25). This blocking of substances also includes the blocking of most antineoplastic therapies due to their large size. Therapies that can make it past the BBB are often not targeted directly to the tumor which leads to the host of treatment-associated negative side effects observed in patients (26).

Immunotherapy has shown tremendous promise for treating cancers residing outside the CNS. However, in brain tumors, immunotherapy trials have had underwhelming results (**Table 1**). Clinical trials such as the CheckMate 143 study were aimed at evaluating the effectiveness of using an ICI (nivolumab) compared to an anti-angiogenic agent (bevacizumab) in patients diagnosed with recurrent GBM (27). The findings for this trial were a disappointment for the field as patients who received nivolumab did not experience a survival benefit compared to bevacizumab-treated (control) patients (27). While the findings from this trial were quite disappointing, subgroup analysis indicated that corticosteroid use at baseline appeared to be unfavorably associated with outcomes in patients that received nivolumab (27). Corticosteroids have previously been shown to negatively impact T-cell function (12, 18, 28). Additionally, it was reported that patients bearing tumors with MGMT promoter methylation had a longer median overall survival compared to patients with unmethylated tumors (27). An additional study that is critical to highlight is a phase 3 study by Lim and colleagues which found that nivolumab in addition to chemoradiation therapy did not improve survival for patients with newly diagnosed GBM with methylated or indeterminate MGMT promoter as compared to patients receiving placebo and chemoradiation (29). The findings from these glioma-targeted ICI-based studies as well as others clearly highlight the need for the field to reconsider whether adjuvant immune checkpoint inhibition is beneficial for patients with GBM.

**Table 1:** Major immune checkpoint inhibitor studies targeting gliomas.

Trial Number	Phase Number	Glioma Type	Treatment	Progression-Free Survival	Overall Survival	Trial Status
**NCT04396860**	Phase III	Glioblastoma	Arm 1: Radiation therapy, Temozolomide Arm 2: Radiation therapy, Ipilimumab, Nivolumab	Not reported due to termination	Not reported due to termination	Suspended
**NCT02017717**	Phase III	Glioblastoma	Arm 1: Nivolumab Arm 2: Bevacizumab	Arm 1: 5.8% at 18 months Arm 2: 8.9% at 18 months	Arm 1: 21.7% at 18 months Arm 2: 21.6% at 18 months	Completed
**NCT02667587**	Phase III	Glioblastoma	Nivolumab, Temozolomide, and Radiotherapy	10.64 months	28.91 months	Active, not recruiting
**NCT02617589**	Phase III	Glioblastoma	Arm 1: Nivolumab, Radiation Therapy Arm 2: Temozolomide, Radiation Therapy	Arm 1: 6.01 months Arm 2: 6.21 months	Arm 1: 13.40 months Arm 2: 14.88 months	Completed
**NCT04817254**	Phase II	Glioblastoma	Arm 1: Nivolumab, Ipilimumab 1mg/kg, Temozolomide Arm 2: Nivolumab, Ipilimumab 3mg/kg, Temozolomide	Not reported at time of review	Not reported at time of review	Recruiting
**NCT04479241**	Phase II	Glioblastoma	Lerapolturev, Pembrolizumab	Not reported at time of review	Not reported at time of review	Active, not recruiting
**NCT03047473**	Phase II	Glioblastoma	Avelumab, with Temozolomide and Radiotherapy	Median: 9.7 months	Median: 15.3 months	Completed
**NCT04396860**	Phase II	Glioblastoma	Arm 1: Radiation Therapy, Temozolomide Arm 2: Radiation Therapy, Ipilimumab, Nivolumab	Not reported due to termination	Not reported due to termination	Suspended
**NCT02798406**	Phase II	Glioblastoma	DNX-2401, Pembrolizumab	Not reported at time of review	Not reported at time of review	Completed
**NCT03673787**	Phase II	Glioblastoma	Ipatasertib, Atezolizumab	Not reported at time of review	Not reported at time of review	Recruiting
**NCT05074992**	Phase II	Glioblastoma	Ipilimumab, Surgery, Chemoradiotherapy	Not reported at time of review	Not reported at time of review	Recruiting
**NCT03430791**	Phase II	Glioblastoma	Arm 1: Nivolumab Monotherapy Arm 2: Nivolumab, Ipilimumab	Results submitted, awaiting quality control review	Results submitted, awaiting quality control review	Terminated
**NCT02550249**	Phase II	Glioblastoma	Nivolumab	Not reported at time of review	Not reported at time of review	Completed
**NCT03367715**	Phase II	Glioblastoma	Nivolumab, Ipilimumab, Short-course radiation therapy	5.92 months	80% at 1 year Median: 16.85 months	Completed
**NCT03890952**	Phase II	Glioblastoma	Arm A: Nivolumab and Bevacizumab, Salvage surgery Arm B: Nivolumab and Bevacizumab, No salvage surgery	Not reported at time of review	Not reported at time of review	Active, not recruiting
**NCT04195139**	Phase II	Glioblastoma	Arm 1: Radiotherapy, Nivolumab, Temozolomide Arm 2: Radiotherapy, Temozolomide	Not reported at time of review	Not reported at time of review	Active, not recruiting
**NCT04145115**	Phase II	Glioblastoma	Ipilimumab, Nivolumab	Not reported at time of review	Not reported at time of review	Recruiting
**NCT03743662**	Phase II	Glioblastoma	Arm 1: Re-irradiation, Bevacizumab, Nivolumab, No surgery Arm 2: Re-irradiation, Bevacizumab, Nivolumab, Re-resection surgery	Not reported at time of review	Not reported at time of review	Active, not recruiting
**NCT03452579**	Phase II	Glioblastoma	Arm 1: Nivolumab, Bevacizumab 10mg/kg Arm 2: Nivolumab, Bevacizumab 3mg/kg	Not reported at time of review	Not reported at time of review	Active, not recruiting
**NCT04704154**	Phase II	Glioblastoma	Regorafenib, Nivolumab	Not reported at time of review	Not reported at time of review	Active, not recruiting
**NCT03718767**	Phase II	IDH-mutant Astrocytoma	Nivolumab	Not reported at time of review	Not reported at time of review	Recruiting
**NCT02794883**	Phase II	Glioblastoma	Arm 1: Tremelimumab Arm 2: Durvalumab Arm 3: Tremelimumab, Durvalumab	Arm 1: 2.746 months Arm 2: 4.356 months Arm 3: 4.913 months	Arm 1: 7.246 months Arm 2: 11.71 months Arm 3: 7.703 months	Completed

Trials evaluating vaccines targeted to GBM have failed as well (10, 30–33). Often, what many of these trials have in common is that these experimental therapies are administered to patients following surgical resection, rather than before. This approach is problematic as surgical resection removes the bulk of the antigen present within the patient which decreases the number of antigenic targets for the immune system. A study by Cloughesy and colleagues reinforces this hypothesis as the research team found that neoadjuvant ICI with anti-PD1 promoted a survival benefit in patients diagnosed with GBM (34).

There are numerous advantages to administering neoadjuvant immunotherapy to patients diagnosed with solid tumors. The first advantage is that this approach allows for a decrease in the size of the tumor allowing for an increased likelihood of achieving a full surgical resection (35). A study by Xu and colleagues found that neoadjuvant immune checkpoint inhibition in patients with squamous cell lung cancer aided in facilitating surgical resection of the disease due to a decrease in tumor size (35). An additional advantage of using a neoadjuvant approach is that one can fully assess whether a patient has the capacity to respond to immunotherapy. Following surgical resection, patients are often immunosuppressed due to receiving agents aimed at reducing cerebral edema (dexamethasone) in addition to receiving cytotoxic chemotherapy and radiation therapy (12, 18). Additionally, the antigen that was present has now been removed due to surgical resection. This combination of factors makes patients poor candidates to receive immunotherapy and limits a clinician’s ability to properly assess whether the patient can respond to immunotherapy. Trials such as NCT04817254 are aimed at designing a novel approach that can assess whether patients with GBM or Gliosarcoma can respond to ICIs.

In this review we will highlight clinical trials and laboratory studies where neoadjuvant ICIs were administered to combat solid tumors residing outside the CNS. In addition to evaluating whether this approach provided an OS and/or PFS benefit, we will also discuss the specific ways this approach enhanced immunological response. We will compare these observations to what has been observed in brain tumor immunotherapy studies as we hope to inspire future studies that evaluate whether neoadjuvant immunotherapy for GBM is an efficacious approach.

## Melanoma

Historically, chemotherapy and interleukin-2 have been the standard of care for advanced melanoma despite their inability to demonstrate a meaningful survival advantage (36). Even with advances in adjuvant immune and targeted therapies, the risk of relapse for higher risk melanomas (stage IIC and IIIB-D) remains high with 10-year OS rates of 24% to 77% (37). The application of neoadjuvant immunotherapy could be viewed as a major disruptor to the current standard of care, with a promise for greater longevity for patients with advanced melanoma. In the last decade, randomized controlled trials using anti-BRAF/MEK targeted therapies such as dabrafenib/tramentinib (DAB + TRAM) or vemurafenib/cometinib, paired with ipilimumab or nivolumab, have demonstrated a dramatic improvement in PFS and OS for unresectable melanoma (38–40). As it stands, there are 52 active, planned, or ongoing interventional trials evaluating neoadjuvant approaches in high-risk melanoma that are registered on clinicaltrials.gov (41). This section will focus on several clinical trials where immunotherapies were administered in a neoadjuvant setting with significant pathological response rates.

In a study of neoadjuvant versus adjuvant ICI in the treatment of clinical stage III melanoma, Song and colleagues reported a neoadjuvant associated 3-year improvement in distant disease-free survival (DDFS) (42). Even after adjusting for ICI agents received, neoadjuvant sequencing remained associated with improved 3-year DDFS as compared to adjuvant therapy. A pathological response was evaluated in 39 patients who received neoadjuvant treatment, with 59% of patients receiving a pathologic partial response and 13% receiving a pathologic complete response. The study enrolled 59 patients, with 18 (31%) receiving adjuvant therapy and 41 (69%) receiving neoadjuvant therapy. Adjuvant therapy was defined as ICI administration after lymph node dissection (LND), while neoadjuvant was defined as administration of one to two cycles of ICI prior to LND, followed by continuation of therapy after surgery. ICI regimens included ipilimumab 3 or 10mg/kg every 3 weeks, pembrolizumab 200mg every 3 weeks, nivolumab 240mg every 2 weeks or 480mg every 4 weeks, or combination ipilimumab 3mg/kg and nivolumab 1mg/kg every 3 weeks in the induction phase followed by nivolumab 240 or 480mg in the maintenance phase.

A phase two study that enrolled patients with high-risk resectable melanoma, evaluated the efficacy and safety of neoadjuvant nivolumab versus combined ipilimumab with nivolumab (43). Despite the trial’s emphasis on monotherapy *vs.* combined neoadjuvant therapy, its findings support the overall efficacy of neoadjuvant immunotherapy administration. The RECIST overall response rates (ORR) was 25% with nivolumab monotherapy and 73% with combined ipilimumab and nivolumab therapy. Additionally, pathologic complete response rates were 25% and 45%, respectively. Although not statistically significant, combination therapy treatment was associated with improved PFS, relapse-free survival (RFS), distant metastasis-free survival (DMFS), and OS over treatment with nivolumab alone. Additionally, improved RFS, DMFS and OS were observed in pathologic complete response patients who received neoadjuvant therapy versus those who did not. However, these results did not reach statistical significance, likely due to small sample size. Consequentially, toxicity rates differed significantly in patients who received neoadjuvant treatment, with reports of grade 3 treatment related adverse events (trAEs) in 8% and 73% of monotherapy and combination therapy patients respectively. A total of 23 patients were enrolled in the trial, with 12 patients randomized to nivolumab therapy monotherapy and 11 patients to combined therapy with ipilimumab and nivolumab. Monotherapy patients received up to four doses of nivolumab 3mgkg^-1^ on weeks 1, 3, 5 and 7, while combined therapy patients received up to three doses of nivolumab 1mgkg^-1^ and ipilimumab 3mgkg^-1^ on weeks 1, 4 and 7.

A phase Ib clinical trial evaluated the feasibility of combined neoadjuvant ipilimumab and nivolumab treatment as compared to an adjuvant regimen for the treatment of melanoma. The study enrolled 20 patients (10: adjuvant; 10: neoadjuvant) with palpable stage III melanoma who were randomized to receive either four courses of combined adjuvant ipilimumab 3mgkg^-1^ and nivolumab 1 mgkg^-1^ every 3 weeks starting at week 6 post-surgery, or two courses of the same regimen, but with neoadjuvant administration every 3 weeks prior to surgery. The study concluded that neoadjuvant-treated patients demonstrated more expanded tumor-resident T-cell clones compared to the adjuvant cohort (44). Additionally, in the neoadjuvant group, 9 of 10 patients were found to have pathological response, 78% of which achieved profound pathological response.

## Melanoma brain metastasis

Melanoma has the highest propensity for the development of brain metastasis among all solid tumors, with studies revealing that up to 44% of all patients with stage IV melanoma develop brain metastases, and 75% of patients have CNS involvement identified at autopsy (45). Until recently, many trials testing the efficacy of newer immunotherapy treatments have excluded patients with melanoma brain metastases (MBM).

A phase 2 clinical trial assessing the efficacy of ipilimumab in patients with melanoma and brain metastases reported activity in some patients, more specifically when metastases were small and asymptomatic (46). Patients older than 16 years with histologically confirmed metastatic melanoma were eligible to be enrolled in this study if they had at least one measurable index brain metastasis of 0.5-3cm in diameter, or two measurable lesions larger than 0.3cm in diameter (46). In the first stage of this study, patients were enrolled into cohort A if they were asymptomatic, to assess the effect of ipilimumab monotherapy on brain and extracranial metastases (46). Patients enrolled in this study received four doses of 10mg/kg of ipilimumab, one every four weeks, and should these patients be clinically stable at week 24, they would then be eligible to receive 10mg/kg of ipilimumab every 12 weeks (46). The primary endpoint for this study was the proportion of patients with complete response, partial response, or stable disease after 12 weeks which was assessed using modified WHO criteria (46). Following the reaching of efficacy parameters, the study proceeded into stage two where patient enrollment into cohort A continued, and patients with symptomatic metastasis controlled with corticosteroids began being enrolled into cohort B (46). The study reported intracranial and extracranial disease control rates of 24% and 27% respectively, in neurologically asymptomatic patients (46). In symptomatic patients, intracranial and extracranial disease control rates were 10% and 5% respectively (46).

Another phase 2 trial with pembrolizumab administration also showed activity in brain metastases in patients with melanoma or NSCLC (47). Patients were enrolled in this study if they were 18 years or older, were diagnosed with melanoma or NSCLC with untreated brain metastases, and had at least one untreated brain metastasis between 5 and 20mm in diameter without associated neurologic symptoms or a need for corticosteroids (47). A total of 18 patients with melanoma and 18 patients with NSCLC were enrolled in this study and patients were given 10mg/kg of pembrolizumab every 2 weeks until progression (47). The primary endpoint for this study was brain metastasis response assessed in all treated patients (47). Brain metastases were assessed by a neuroradiologist with unidimensional evaluation using Response Evaluation in Solid Tumors (RECIST) criteria (version 1.1) (47). Findings from this study were encouraging as a brain metastasis response rate of 22% and 33% was observed in melanoma and NSCLC patients respectively (47).

Finally, a combined nivolumab and ipilimumab phase 2 study also showed greater efficacy in patients with asymptomatic melanoma with brain metastases than prior monotherapy studies (48). In this study, patients aged 18 years or older with measurable melanoma brain metastases that were 0.5-3.0cm in diameter were enrolled into either cohort A if they were asymptomatic, had an Eastern Cooperative Oncology Group (ECOG) performance status of 0 or 1, no neurologic symptoms or baseline corticosteroid use, or cohort B if they were symptomatic, had an ECOG performance status of 0-2 with stable neurological symptoms (48). Patients in cohort B could be receiving low-dose dexamethasone (48). Patients in both cohorts received nivolumab 1mg/kg plus ipilimumab 3mg/kg every three weeks for four doses, followed by nivolumab 3mg/kg every 2 weeks for up to 2 years until either disease progression or unacceptable toxicity (48). The primary endpoint for this study was intracranial clinical benefit rate (complete responses, partial responses, or stable disease lasting 6 months or more) assessed in all patients that were treated (48). Response was determined *via* radiographic assessment that was performed every 6 weeks for the first year of study enrollment, and then every 12 weeks thereafter, until documented disease progression, using RECIST (version 1.1) criteria (48). The study reported an intracranial clinical benefit of 57.4% and 16.7% for neurologically asymptomatic and symptomatic patients respectively (48).

## Non-small cell lung cancer

Many clinical trials have indicated that neoadjuvant immune checkpoint inhibition for patients with NSCLC could be a promising treatment modality. Few trials exist that have evaluated the efficacy of adjuvant ICIs for patients with NSCLC, let alone trials that have compared neoadjuvant ICI administration to adjuvant ICI administration. Given these findings, this section will focus on clinical trials where ICIs were administered in a neoadjuvant setting.

In a phase 2 clinical trial that evaluated the efficacy of administering neoadjuvant atezolizumab to patients with resectable NSCLC, 21% of the patient population achieved a major pathological response and 7% of patients achieved a complete pathological response (49–51). These findings were correlated with a major pathological response rate of 33% in 50% of patients expressing the highest PD-L1 protein level (49–51). The 50% of patients expressing the lowest PD-L1 protein levels had a major pathological response rate of 11% (49–51). NCT02259621 is another study that evaluated the efficacy of administering neoadjuvant checkpoint inhibition to patients with stage IB-IIIA NSCLC. The trial enrolled 15 patients who received 3 mg/kg of nivolumab and 1 mg/kg of ipilimumab intravenously 6 weeks prior to surgical resection (52). 3 mg/kg of nivolumab was then given again 4 and 2 weeks preoperatively. Due to toxicity the study was terminated early (52). However, of the six patients who underwent surgical resection three patients were alive with no recurrence of disease, two patients experienced a recurrence, and one patient died postoperatively of acute respiratory distress syndrome (52).

The NADIM trial enrolled patients with resectable stage IIIA NSCLC. These patients underwent three cycles of neoadjuvant nivolumab and chemotherapy prior to surgery, and one year of adjuvant nivolumab monotherapy following surgery. Following tumor resection, it was observed that 85.4% of patients survived without any recurrence of their disease (53). Additionally, down-staging occurred in approximately 90% of the cases within this study (53). Interestingly, the team notes that there were no significant associations identified between any clinical or molecular parameters analyzed at the time of diagnosis and a patient’s pathological response. However, the research team did observe that a PD-L1 tumor proportion score of 25% or more was associated with major or complete pathological response (53). However, this metric was insufficiently sensitive as 58% of patients with a PD-L1 tumor proportion score of less than 25% also demonstrated major or complete pathological responses (53). This trial enrolled a limited number of patients which may have impacted the ability to identify significant associations between clinical or molecular parameters identified at the time of diagnosis and pathological response.

Recently, a phase 2 study called the NEOSTAR trial was completed. The trial enrolled patients with surgically resectable NSCLC. This study sought to evaluate whether there was a survival benefit associated with administering neoadjuvant nivolumab and ipilimumab as opposed to administering nivolumab alone. This trial enrolled 44 eligible patients with 23 patients being assigned to the nivolumab monotherapy arm and 21 patients being assigned to the nivolumab plus ipilimumab cohort (54). Patients received doses of either nivolumab alone (3 mg/kg) or nivolumab and ipilimumab (3 mg/kg and 1 mg/kg respectively) at days 1, 15 and 29 (54). This regimen was followed by surgical resection which was planned for at least 21 days after and within 42 days of receiving the first dose of nivolumab. The research team found that 38% of patients in the nivolumab plus ipilimumab cohort achieved a MPR of 38% as compared to a 22% MPR in the nivolumab monotherapy cohort (54). In patients that underwent surgical resection, the MPR for the nivolumab plus ipilimumab cohort was 50% as compared to 24% for the nivolumab monotherapy cohort (54). Patients in the nivolumab plus ipilimumab cohort had higher complete pathological response rates (38% as compared to 10%), less viable tumor, and a greater number of effector, tissue resident memory, and effector memory T-cells (54).

A final study that is critical to highlight is a phase 3 trial led by Forde and colleagues which found that neoadjuvant nivolumab plus chemotherapy had promising findings in patients with NSCLC. In this study patients with stage IB to IIIA resectable NSCLC were randomly assigned to receive nivolumab (360mg) plus platinum-based chemotherapy or platinum-based chemotherapy alone every three weeks for three cycles (55). Surgery was planned to take place within 6 weeks following the completion of neoadjuvant treatment (55). Following surgery, patients in either the neoadjuvant nivolumab plus chemotherapy group, or the neoadjuvant chemotherapy alone group could receive up to four cycles of adjuvant chemotherapy, radiotherapy, or both (55). There were two primary endpoints for this trial, the first being event-free survival (55). The second primary endpoint was pathological complete response (55). In this trial, 352 patients received treatment, with 176 patients receiving neoadjuvant nivolumab plus chemotherapy, and 176 patients receiving chemotherapy alone (55). Findings from this trial were encouraging as the median event-free survival was 31.6 months for patients in the nivolumab plus chemotherapy group as opposed to a median event-free survival of 20.8 months for patients in the chemotherapy alone group (55). The percentage of patients with a pathological complete response was 24% for patients in the nivolumab plus chemotherapy group, and 2.2% for patients in the chemotherapy alone group (55). These findings highlighted that neoadjuvant nivolumab plus chemotherapy resulted in significantly longer event-free survival and a higher percentage of patients with a pathological complete response.

Pembrolizumab is an additional ICI that has been approved for the treatment of advanced NSCLC however there are few trials that have evaluated its usefulness in a neoadjuvant setting. In the NEOMUN trial, patients with stage IIA-IIIA NSCLC will receive two cycles of pembrolizumab as a neoadjuvant immunotherapy and clinical and pathological tumor response will be assessed (56).

## Breast cancer

Breast cancer is the most common cause of cancer in women worldwide. The development of new immunotherapies to treat cancer, such as ICIs, have shown success in treating cancers such as malignant melanomas, non-small cell lung cancer, colon, and rectal cancer. While these types of treatments have shown efficacy in other types of cancer, breast cancer pathogenicity is somewhat unique in that it’s considered to be immunologically “cold,” thus immunotherapeutic approaches to treating breast cancer still have many unresolved issues (57). Immunotherapy, when provided in the neoadjuvant setting, is expected to be used as a new treatment modality when treating breast cancer, especially for phenotypes which have high immunogenicity, such as triple negative breast cancer (TNBC). Here, we will review clinical trials where neoadjuvant immunotherapeutic treatments were administered to patients with breast cancer.

A randomized phase 2 I-SPY2 trial examined the efficacy of neoadjuvant treatment that included pembrolizumab, on participants with early-stage, high-risk, ERBB2 (formerly HER2)-negative breast cancer. In the neoadjuvant setting, drug efficacy can be evaluated using pathological complete response (pCR) as a survival endpoint, which is defined as the absence of invasive tumor in breast and regional lymph nodes at the time of surgery (58). Based on accumulated results, pCR after neoadjuvant treatment correlates significantly with the PFS and OS rate (59). Out of the 250 women included in the final analysis, 181 were randomized to receive standard NACT therapy (control arm), which included paclitaxel for 12 weeks, in addition to 4 cycles of doxorubicin plus cyclophosphamide every 2 to 3 weeks (AC). 69 participants in the intervention group received the standard NACT therapy in addition to neoadjuvant pembrolizumab every 3 to 4 cycles concurrently with paclitaxel. 40 participants were hormone receptor (HR) positive and 29 were triple-negative. Estimated pCR rates showed an increase in pCR for all cohorts of pembrolizumab *vs.* control, (44% *vs.* 13%) in ERBB2-negative, (30% *vs.* 13%) in HR-positive/ERBB2-negative, and (60% *vs.* 22%) in triple-negative. Adverse reactions to the intervention included thyroid abnormalities (13%) and adrenal insufficiency (8.7%). As stated, pCR significantly correlates with survival rate and this study showed that participants with pCR following pembrolizumab plus chemotherapy had high 3-year event-free survival rates (93% at 2.8 years’ median follow-up). Findings from this study showed that the addition of pembrolizumab to standard neoadjuvant chemotherapy more than doubled pCR compared to chemotherapy alone for hormone receptor-positive/ERBB2-negative and triple-negative breast cancer (60).

A phase 3 KEYNOTE-522 clinical trial similarly evaluated the benefit of adding neoadjuvant pembrolizumab to neoadjuvant chemotherapy for participants diagnosed with early TNBC. Participants diagnosed with untreated stage 2 or stage 3 TNBC were randomly assigned to receive either 4 cycles of neoadjuvant chemotherapy (paclitaxel and carboplatin) plus placebo every three weeks (control), or 4 cycles of pembrolizumab in addition to paclitaxel and carboplatin every 3 weeks. Both groups also received doxorubicin-cyclophosphamide or epirubicin-cyclophosphamide. Following surgery, participants then received 9 cycles of adjuvant pembrolizumab or chemotherapy every 3 weeks. After subsequent analysis, it was reported that out of the 602 participants who were randomized in the study, the pCR rates for the pembrolizumab-chemotherapy group and the chemotherapy-placebo groups were 64.8% and 51.2%, respectively. The estimated treatment difference was calculated to be 13.6%. After follow-up, 7.4% of participants in the pembrolizumab-chemotherapy group and 11.8% of participants in the chemotherapy-placebo group were found to have either disease progression post-surgery, distal or local recurrence of disease, a second primary tumor, or died from any cause. Findings from this study demonstrated that among participants diagnosed with early, untreatable stage 2/3 TNBC, the pCR was significantly higher among participants in the cohort who received neoadjuvant pembrolizumab plus neoadjuvant chemotherapy compared to those who received neoadjuvant chemotherapy plus neoadjuvant placebo (61).

A randomized, double-blind phase 3 clinical trial, IMpassion031, compared the efficacy and safety of the drug atezolizumab *vs.* placebo in combination with nab-paclitaxel followed by doxorubicin plus cyclophosphamide for patients with TNBC. Participants of the study had to be 18 years or older and diagnosed with previously untreated stage 2 or stage 3 TNBC. Participants received either neoadjuvant IV atezolizumab plus chemotherapy every 2 weeks or received neoadjuvant placebo plus chemotherapy. The chemotherapy regimen consisted of nab-paclitaxel every week for 12 weeks, followed by doxorubicin and cyclophosphamide every 2 weeks for 8 consecutive weeks then followed by surgery. Out of the 333 eligible participants of the study, 165 were randomly assigned to receive atezolizumab plus chemotherapy and 168 were assigned to receive placebo plus chemotherapy. The median follow-up for the atezolizumab plus chemotherapy group was 20.6 months and 19.8 months for the group who received placebo plus chemotherapy. A pCR rate of 58% was reported in 95 patients who received the atezolizumab plus chemotherapy and a pCR rate of 41% was reported in 69 participants who received the placebo plus chemotherapy. The rate difference between the two groups was reported as 17%. For the population of participants that were PD-L1 positive, pCR was reported as 69% in 53 out of the 77 participants that received the atezolizumab plus chemotherapy. For the PD-L1 positive participants who received placebo plus chemotherapy, 37 out of the 75 participants reported a pCR rate of 49%. The rate difference between the two groups was reported as 20%. Findings from this study demonstrated that in patients with previously untreated, diagnosed early-stage TNBC, neoadjuvant treatment with atezolizumab in combination with chemotherapy significantly improved pCR rates compared to patients who received placebo in combination with chemotherapy (62).

## Colon and rectal cancer

Numerous strategies have been utilized to activate cancer immunity in colorectal cancer (CRC) with major modalities of immunotherapy including monoclonal antibodies, ICIs, cancer vaccines, adoptive cell therapies, and bispecific T-cell engagers (63). Implementation of immunotherapy has long been a desired goal for treating CRC due to its tailorability and promising potential for inducing longer-term forms of immune surveillance, theoretically decreasing risks of future recurrence of disease (63). Even though much of the evidence supporting the use of ICIs is most abundant in cases of metastatic treatment-refractory cases of gastric cancer and hepatocellular carcinoma (64); these findings have highlighted the potential of using ICIs for the treatment of CRC. Previous studies found that response rates were independent of biologic marker status, such as microsatellite instability-high (MSI-H)/deficient MisMatch Repair (dMMR) or programmed cell death-ligand 1 (PD-L1) expression (63). Despite initial studies of ICIs demonstrating limited activity in unselected CRC patients, this section will focus on several clinical trials where ICIs were administered in a neoadjuvant setting allowing for significant pathological response rates.

In a phase three clinical trial that evaluated efficacy of administering neoadjuvant pembrolizumab to patients with MSI-H advanced CRC (65), patients experienced a median PFS of 16.5 months compared to 8.2 months when chemotherapy alone was administered. Correlative to these findings was an increase in overall response (complete or partial response as evaluated with Response Evaluation Criteria in Solid Tumors), with 43.8% of patients in the pembrolizumab group and 33.1% in the chemotherapy group achieving an overall response. Of the patients with an overall response, 83% in the pembrolizumab group, compared with 35% in the chemotherapy group, had continual responses at 24 months, changing the standard of care for metastatic CRC with dMMR (66). The trial enrolled 307 patients who received 200 mg of pembrolizumab every three weeks or chemotherapy (5-fluorouracil-based therapy with or without bevacizumab or cetuximab) every two weeks. The study allowed patients in the chemotherapy cohort to subsequently convert to pembrolizumab therapy with any sign of disease progression.

A phase two study that enrolled patients with metastatic MSI-H/deficient MisMatch Repair (dMMR) CRC, evaluated the two-year long-term efficacy and safety of neoadjuvant nivolumab plus low-dose ipilimumab (67). Patients were treated with nivolumab every two weeks plus low dose ipilimumab every six weeks until disease progression. An objective response rate and disease control rate of 69% and 84%, respectively, were observed, with a complete response rate of 13%. The team noted that even though a median duration of response was not reached, 74% of responders had ongoing responses at data cutoff. Interestingly, a *post hoc* analysis of 14 patients who had discontinued treatment was also performed and ten remained progression-free. Additionally, at the end of the 24-month period, the team noted a median PFS and OS of 74% and 79% respectively. A consequential finding of this study was that regardless of baseline demographic and tumor characteristics, including *BRAF* or *KRAS* mutation status, clinical benefit was still observed (67).

Another study that is important to highlight is a phase two study that investigated the activity of a neoadjuvant nivolumab/ipilimumab regimen in both dMMR and proficient MisMatch Repair (pMMR) early-stage (Stage I-III) resectable colon adenocarcinomas (68). Patients received combination treatment of ipilimumab and nivolumab on day one (1mgkg^-1^ and 3mgkg^-1^, respectively) as well as a dose of nivolumab (3mgkg^-1^) on day fifteen. Following radical tumor resection at four weeks post initial treatment, it was observed that all (100%) patients with dMMR tumors showed pathological response *via* tumor regression. 95% of those patients had a major pathological response (MPR, <10% residual viable tumor). Additionally, 27% of patients with pMMR tumors showed a pathological response, with 50% of those showing complete pathological response. Changes in the microenvironments were also noted for dMMR tumors, with a significant increase in CD8+ and CD3+ T-cell infiltrates, as well as IFN-γ scores compared to pretreated biopsies (68). At the time of resection, these tumors also noted a significant increase in tertiary lymphoid structures (TLS), which have been found to harbor most PD-1+ tumor infiltrating lymphocytes (TILs) in lung cancer (69). Forty patients were enrolled in this trial with 21 dMMR and 20pMMR tumors (one patient had both pMMR and dMMR colon cancer).

Finally, results from the NICHE-2 study, a study treating patients with deficient mismatch repair (dMMR) colon cancer with neoadjuvant nivolumab and ipilimub has shown promising findings. Specifically, in this study, patients with non-metastatic dMMR colon cancer were treated with one dose of ipilimumab (1mg/kg) and two doses of nivolumab (3mg/kg) followed by surgery (70). The two primary endpoints for this study were safety, and 3-year disease-free survival (DFS) (70). A total of 112 patients were treated in this study with radiographic assessment performed at baseline revealing 89% of tumors to be stage III, 77% being high-risk stage III and 64% being considered T4 tumors (70). A major pathological response was observed in 95% of patients, and complete response was observed in 67% of patients (70). The findings from this trial were some of the first to clearly indicate that there is a strong potential for neoadjuvant immunotherapy to become the standard of care for patients with dMMR colon cancer (70).

## Brain tumors

There have been numerous studies where patients with brain tumors have undergone adjuvant immune checkpoint inhibition. Many of these trials have failed which have led many to believe that ICIs may not be a useful treatment for brain tumors, especially GBM (27, 71–73). While there are numerous contributors that may have led to the failure of these trials some of the most likely reasons include, patients receiving corticosteroids prior to study enrollment or while on the study, and tumor resection prior to treatment initiation.

One of the first studies to explore the effectiveness of administering neoadjuvant immunotherapy to patients with brain tumors was a study by Cloughesy and colleagues which evaluated whether patients with recurrent GBM experience a survival benefit when receiving neoadjuvant anti-PD1 prior to undergoing surgical resection (34). Patients with recurrent GBM receiving neoadjuvant anti-PD1 were compared to those with recurrent GBM receiving adjuvant anti-PD1 following surgical resection. There were 35 total patients enrolled in this study with 16 patients being assigned to the neoadjuvant cohort and 19 patients being assigned to the adjuvant cohort (34). Following patient enrollment and cohort assignment, those patients assigned to the neoadjuvant cohort received 200 mg intravenous infusions of pembrolizumab 14 ± 5 days prior to surgical resection. Following resection, patients in both cohorts received 200 mg intravenous infusions of pembrolizumab every three weeks until tumor progression or until the occurrence of an adverse event requiring treatment discontinuation.

The research team found that patients receiving neoadjuvant anti-PD1 had a median OS of 13.7 months and those receiving adjuvant anti-PD1 had a median OS of just 7.5 months. Additionally, PFS was enhanced for those receiving neoadjuvant therapy as these patients had a median PFS of 3.3 months as compared to those receiving adjuvant therapy who had a median PFS of 2.4 months (34). Outside of a survival benefit, it was also noted that patients receiving neoadjuvant therapy experienced an increase in T-cell and interferon-gamma related gene expression within their tumors as well as a downregulation of cell cycle-related gene expression (34). This was not observed in patients receiving adjuvant therapy. It was also observed that those undergoing neoadjuvant treatment had an enhanced clonal expression of T-cells as well as a decreased expression of PD-1 on T-cells within the peripheral blood (34). This study was one of the first of its kind and highlighted the promise of not only using immunotherapy to treat GBM, but also the potential of administering this therapy neoadjuvantly.

Since the study by Cloughesy and colleagues there have been few studies that have explored the benefit of administering neoadjuvant immunotherapy to patients with GBM. A study by Prins and colleagues found that neoadjuvant anti-PD1 induces T-cell and cDC1 activation in patients with recurrent GBM however, this treatment modality failed to overcome immunosuppressive tumor associated macrophages that have a large presence in the TME of patients with GBM (74). A clinical trial by a group of collaborators at Duke University (NCT04434560) explored the efficacy of administering neoadjuvant ICIs to patients with brain metastases however this trial was terminated due to poor enrollment.

Few other studies have been performed to date that explore the efficacy of administering neoadjuvant immunotherapy to patients with GBM. Additionally, studies that have been performed have only evaluated whether there is a survival benefit for patients with *recurrent* GBM. This is problematic as patients with recurrent GBM are typically immunosuppressed as they have undergone treatment with corticosteroids, cytotoxic chemotherapy as well as radiation therapy. More studies are needed that explore whether there is an even larger benefit to treating patients with their initial GBM with neoadjuvant immunotherapy as these patients have not undergone the immunosuppressive milieu of treatments that most GBM patients undergo.

While some success has been observed in studies targeting ICIs to brain metastases, few ICI studies have demonstrated success when adjuvant ICIs were targeted to GBM. One of the reasons for this lack of success could be differences in the tumor microenvironment between brain metastases and GBM. While there is a paucity of literature that clearly outlines differences in the tumor microenvironment of brain metastases compared to that of GBM, some studies have shown that both microenvironments are characterized by high numbers of myeloid cells that are associated with an immunosuppressive phenotype (75, 76). However, a key difference in the microenvironments of brain metastases and GBM may involve spatial heterogeneity, as a recent study by Schaettler and colleagues found that GBMs display more spatial heterogeneity at the genomic and neoantigen levels as compared to brain metastases (77). Additionally, the spatial diversity that was observed in this study was recapitulated in T-cell clone distribution as some GBMs possessed highly expanded yet spatially restricted clonotypes as compared to less spatially restricted clonotypes for brain metastases (77). These findings clearly indicate that far more research is needed to fully understand differences in the tumor microenvironments of GBMs and brain metastases as these differences may highlight areas of vulnerability that can be exploited when targeting neoadjuvant immunotherapies to GBM.

## Conclusion

In this review we have highlighted several clinical studies that have demonstrated the effectiveness of administering neoadjuvant ICIs to patients diagnosed with tumors of the skin, lung, breast, as well as the colon and rectum (see **Table 2**). We selected these tumors due to their comparability to GBM, as these tumors are often classified as “immunologically cold”, grow aggressively, and are often accompanied by a poor patient prognosis. In most of the studies highlighted throughout this review neoadjuvant immune checkpoint inhibition had a profound impact on OS as well as PFS, as this treatment modality allowed for an increased patient immune response. Beyond the malignancies treated with neoadjuvant immune checkpoint inhibition that we have highlighted in this manuscript, promising results have also been observed in patients diagnosed with squamous-cell carcinoma (78). In a phase 2 trial led by Gross and colleagues, patients with stage II, III, or IV cutaneous squamous-cell carcinoma received cemiplimab at a dose of 350mg every 3 weeks for up to four doses prior to undergoing surgery with curative intent (78). A pathological complete response was observed in 51% of patients that received neoadjuvant cemiplimab, and an objective response on imaging was observed in 68% of patients (78).

**Table 2:** Neoadjuvant immune checkpoint inhibitor studies targeting non-CNS and CNS cancers.

Trial Number	Phase	Cancer Type	Neoadjuvant Treatment	Adjuvant Treatment	Progression-free Survival	Overall Survival	Trial Status
**NCT01274338**	Phase III	Melanoma Stage III	Arm 1A: Ipilimumab	Arm 2A: Ipilimumab	Arm 1A-C: 62% at 36 months	Not reported at time of review	Recruiting
			Arm 1B: Ipilimumab + Nivolumab	Arm 2B: Ipilimumab + Nivolumab	Arm 2A-C: 31% at 36 months		
			Arm 1C: Pembrolizumab or Nivolumab	Arm 2C: Pembrolizumab or Nivolumab			
		
**NCT02519322**	Phase II	Melanoma Stage IIIB-IV	Arm 1: Nivolumab	All patients undergoing surgery were offered 13 doses of Nivolumab post-surgery	Arm 1 patients: 58% at 22.6 months	Arm 1 patients: 76% at 22.6 months	Active, not recruiting
			Arm 2: Nivolumab + Ipilimumab		Arm 2 patients: 82% at 17.2 months	Arm 2 patients: 100% at 24 months	
			Both arms completed cycles prior to surgery				
**NCT00623766**	Phase II	Melanoma with Brain Metastases	N/A	Arm 1: Ipilimumab	Arm 1: 1.4 months	Arm 1: 26% at 36 months	Completed
				Arm 2: Ipilimumab + corticosteroid	Arm 2: 1.2 months	Arm 2: 10% at 36 months	
**NCT02437279**	Phase I	Melanoma Stage III	Arm 1: Nivolumab + Ipilimumab	Arm 2: Nivolumab + Ipilimumab after Complete Lymph Node Dissection	Arm 1: 80% at 25.6 months	Arm 1: 90% at 25.6 months	Active, not recruiting
			Complete Regional Lymph Node Dissection at Week 6		Arm 2: 60% at 25.6 months	Arm 2: 60% at 25.6 months	
**NCT02927301**		Non-Small Cell Lung Cancer Stage IB-IIIB	Atezolizumab	N/A	Not a reportable outcome	Not a reportable outcome	Active, not recruiting
	Phase II		Resection at day 40 +/- 10 days				
**NCT02259621**	Phase II	Non-Small Cell Lung Cancer Stage IB-IIIA	Nivolumab + Ipilimumab	All patients offered standard postoperative adjuvant chemotherapy+/- radiation	33% at 25 months	Not a reportable outcome	Recruiting
**NCT03081689**	Phase II	Non-Small Cell Lung Cancer Stage IIIA	Nivolumab + Paclitaxel + Carboplatin	Adjuvant treatment commenced 3-8 weeks post-surgery	77.1% at 24 months	89.9% at 24 months	Recruiting
			Surgical resection planned 42-49 days after 1^st^ day of third treatment cycle				
**NCT03158129**	Phase II	Non-Small Cell Lung Cancer Stage I-IIIA	Arm 1: Nivolumab	Standard adjuvant chemotherapy and/or postoperative radiation allowed at the discretion of treating physician	Median PFS was not reached	Median OS was not reached	Recruiting
			Arm 2: Nivolumab + Ipilimumab				
			Surgical resection at least 3 weeks and within 6 weeks post last dose of nivolumab				
**NCT03197467**		Non-small Cell Lung Cancer Stage II/IIIA	Pembrolizumab	Adjuvant chemotherapy will be recommended according to the current national and international guidelines	Not reported at time of review	Not reported at time of review	Active, not recruiting
	Phase II		Surgical resection Day 50-60				
**NCT03036488**	Phase III	Triple Negative Breast Cancer	Arm 1: Chemotherapy + Paclitaxel + Carboplatin + Doxorubicin or Epirubicin-Cyclophosphamide	Adjuvant pembrolizumab or chemotherapy was given every 3 weeks for up to 9 cycles	Arm 1: 85.3% at 18 months	Not reported at time of review	Active, not recruiting
			Arm 2: Pembrolizumab + Paclitaxel + Carboplatin + Doxorubicin or Epirubicin-Cyclophosphamide		Arm2: 91.3% at 18 months		
			Surgical resection 3-6 weeks after last cycle				
**NCT03197935**	Phase III	Triple Negative Breast Cancer	Arm 1: Nab-paclitaxel + Doxorubicin + Cyclophosphamide	Arm 1: Nab-paclitaxel + Doxorubicin + Cyclophosphamide	Not reported at time of review	Not reported at time of review	Active, not recruiting
			Arm 2: Atezolizumab + Nab-paclitaxel + Doxorubicin + Cyclophosphamide	Arm 2: Atezolizumab			
			Surgical resection followed				
**NCT01042379**	Phase II	*ERBB2*-Negative Breast Cancer	Arm 1: Paclitaxel + Doxorubicin + IV Cyclophosphamide	Adjuvant treatment was not mandated by this trial	Not reported at time of review	Not reported at time of review	Recruiting
			Arm 2: Paclitaxel + Doxorubicin + IV Cyclophosphamide + Pembrolizumab				
			Definitive surgery followed				
**NCT02563002**	Phase III	Stage IV Colorectal Carcinoma	Arm 1A: Oxaliplatin + Leucovorin + 5-Fluoropyrimidine	N/A	Arm 1A-F: 18.6% at 24 months	Arm1A-F: 52% at 32.4 months	Active, not recruiting
			Arm 1B: Oxaliplatin + Leucovorin + 5-Fluoropyrimidine + Bevacizumab		Arm 2: 48.3% at 24 months	Arm 2: 63% at 32.4 months	
			Arm 1C: Oxaliplatin + Leucovorin + 5-Fluoropyrimidine + Cetuximab				
			Arm 1D: Irinotecan + Leucovorin + 5-Fluoropyrimidine				
			Arm 1E: Irinotecan + Leucovorin + 5-Fluoropyrimidine + Bevacizumab				
			Arm 1F: Irinotecan + Leucovorin + 5-Fluoropyrimidine + Cetuximab				
			Arm 2: Pembrolizumab				
		
**NCT04008030**	Phase III	Metastatic Colorectal Cancer	Nivolumab + Ipilimumab	N/A	Median PFS was not reached (74% at 24 months)	Median OS was not reached (79% at 24 months)	Recruiting
**NCT03026140**	Phase II	Early-Stage Colon Cancer	Arm 1: Nivolumab	N/A	Not reported at time of review	Not reported at time of review	Recruiting
			Arm 2: Nivolumab + Ipilimumab				
			Arm 3: Nivolumab + Ipilimumab + Celecoxib				
			Surgery resection at 6 weeks				
**NCT04201873**	Phase I	Glioblastoma	Arm 1: Pembrolizumab	Arm 2: Pembrolizumab	Arm 1: 99.5 days	Arm 1: 417 days	Recruiting
					Arm 2: 72.5 days	Arm 2: 228.5 days	
**NCT04434560**	Phase II	Brain Metastases	Pembrolizumab & Ipilimumab	Standard postoperative adjuvant treatment	Not reported due to termination	Not reported due to termination	Terminated
			Surgical resection 7 days post treatment				

While results have been encouraging for treating cancers outside the brain with neoadjuvant ICIs, very few studies have evaluated the effectiveness of administering neoadjuvant ICIs to patients with GBM. We believe this is an area in need of further investigation as currently, GBM patients receive ICIs following surgery when the antigen has been removed from the cranial compartment. This antigen removal can be a barrier to the induction of a robust anti-tumor immune response due to decreased antigen load.

Additionally, GBM patients are often immunosuppressed and are therefore not best positioned to fully respond to ICIs (79, 80). Studies by Fecci and colleagues observed that patients with GBM have T-cell sequestration in the bone marrow due to the loss of the S1P1 receptor, while Giles and colleagues describe how dexamethasone (a corticosteroid commonly given to GBM patients to control cerebral edema) induces immunosuppression (18, 81). These are just some of the many ways that GBM patients become immunosuppressed throughout their tumor course which ultimately contribute to treatment failure (10, 12, 28). There may be major benefits to neoadjuvant immune checkpoint inhibition as this is when patients may be best positioned to respond to treatment as they are earlier in their treatment course and have not undergone surgical resection as surgery is known to create an anti-inflammatory tumor microenvironment. Given the large number of ICI clinical studies that have failed in neuro-oncology, phase 0 studies may be beneficial in evaluating whether neoadjuvant ICI is beneficial for patients with GBM as these studies are often first in human, enroll a small number of patients (lowering study-associated expenses), and can help investigators determine (*via* blood and/or tissue analysis) whether neoadjuvant ICI better primes the immune system as compared to adjuvant ICI.

In **Table 3** we highlight several other benefits for the neoadjuvant administration of immune checkpoint inhibitors such as a reduction in tumor burden prior to surgery, the assessment of a pathologic immune response to immunotherapy, in addition to other benefits. We believe that the time is now for the field of neuro-oncology to begin evaluating whether there is increased benefit to administering ICIs prior to surgery as this may be the time the patient is most likely to respond to treatment and experience a survival benefit.

**Table 3:** Summary of the potential benefits of neoadjuvant administration of ICIs.

Benefits of Neoadjuvant Immunotherapy	Citation
Reduces tumor burden prior to surgery, allowing for more optimal resection	(82)
Allows for the ability to fully assess pathologic response to immunotherapy	(83)
Allows for the utilization of treatment response as surrogate outcome markers for relapse-free and overall survival	(82)
Allows for a greater chance of immune to tumor microenvironment interaction, resulting from preservation of the lymphatic system	(84)
Induces a stronger immune response (tumor specific CD8^+^ T-cells) against a higher tumor antigen load	(85)
More cost effective than adjuvant treatment as a result of fewer treatments needed	(84)
Can attenuate the immunosuppressive effects of surgery, such as the systemic release of glucocorticoids, which suppress T-cell proliferation and induce apoptosis of naïve T-cells	(86)

## Author contributions

SF, CD, JB, and AD performed a review of the literature and wrote the manuscript. XZ and CH provided their expertise on the clinical aspects of the manuscript. GK conceptualized and supervised the writing of the manuscript. All authors contributed to the article and approved the submitted version.
